# Self-inflicted stab injury with abdominal evisceration: A case report

**DOI:** 10.1016/j.ijscr.2021.106543

**Published:** 2021-11-02

**Authors:** Anuj Parajuli, Aakash Mishra, Roshan Ghimire, Suman Kumar Shrestha

**Affiliations:** a Department of Surgery, Kathmandu Medical College Teaching Hospital, Kathmandu, Nepal; b Kathmandu Medical College Teaching Hospital, Kathmandu, Nepal

**Keywords:** Self-inflicted injury, Abdominal evisceration, Colon injury, Schizoaffective disorder

## Abstract

**Introduction and importance:**

Self-inflicted abdominal stab injury with an intention of self-harm is uncommon. Moreover, self-inflicted injury leading to avulsion of the colon has rarely been reported in the literature. We report a case of a 42-years-female with schizoaffective disorder who presented with self-inflicted stab injury on the abdomen resulting in abdominal evisceration.

**Presentation of case:**

A 42-years-female with schizoaffective disorder (F25) for 10 years presented to the emergency department with multiple, self-inflicted injuries on the abdomen. A large free portion of the omentum and segment of the bowel were brought in a plastic carry bag. Examination revealed multiple transverse hesitation cuts in the epigastrium and a single deep penetrating transverse cut resulting in the evisceration of the omentum and colon. Intra-operatively, avulsion of a large portion of the greater omentum and missing segment of the mid transverse colon was observed. The patient underwent an immediate abdominal exploration and side-to-side colo-colic anastomosis along with diversion ileostomy. At three months following primary surgery, ileostomy closure was done.

**Conclusion:**

Patients with schizophrenia spectrum psychosis are at risk of self-harm and in our case a schizoaffective patient presented with self-inflicted injuries that required an emergency abdominal exploration and repair. This case highlights a multi-disciplinary approach for the management of these cases and mandates clinicians and caregivers to be more vigilant to restrict injuries in the future.

## Highlights

• Self-inflicted abdominal stab injuries resulting in segmental transection of the transverse colon is rare.
• Self-inflicted injuries in psychotic patients can occur without any warning signs and despite treatment compliance.
• Severe injuries on the abdomen can be life threatening and requires emergent surgical management.

## 1 Introduction

Self-infliction injuries are acts of deliberately harming one's own body with or without an intention of committing suicide [1]. They may be associated with risk factors like adolescence, negative life events, history of childhood sexual abuse, personal and environmental factors, low emotional expressivity, and low self-esteem [2]. Self-infliction injuries may include injuries to any part of the body; nevertheless not many cases related to colon injuries have been recorded. Colon injury has been reported in 15% to 39% of patients with penetrating abdominal injuries and the ascending and transverse colon are the most commonly injured segments [3]. The mechanism of injury to the colon commonly involves penetrating injuries as stab and gunshot injury, blunt trauma, and de-vascularization injury secondary to avulsion of the supporting mesentery. The treatment choices available for colon injuries are exploration with primary repair (suture repair or resection and anastomosis) and diversion with stoma formation [4]. This work has been reported in line with the SCARE criteria [5].

## 2 Case presentation

A 42-years-female with schizoaffective disorder (F25) for 10 years presented with multiple transverse hesitation cuts in the epigastrium with a single deep penetrating transverse cut resulting in the evisceration of the omentum and colon.

At presentation in the emergency room (ER), she was conscious, pale with a blood pressure of 90/60 mmHg, pulse 120 beats per minute, and respiratory rate of 22 breaths per minute. On examination, multiple transverse hesitation cuts on the epigastrium and large transverse penetrating cut measuring 10 × 3 cm in the epigastrium with evisceration of omentum and parts of the bowel were seen. A large free portion of the omentum and segment of the bowel was brought along in a plastic carry bag.

**Fig. 1:**
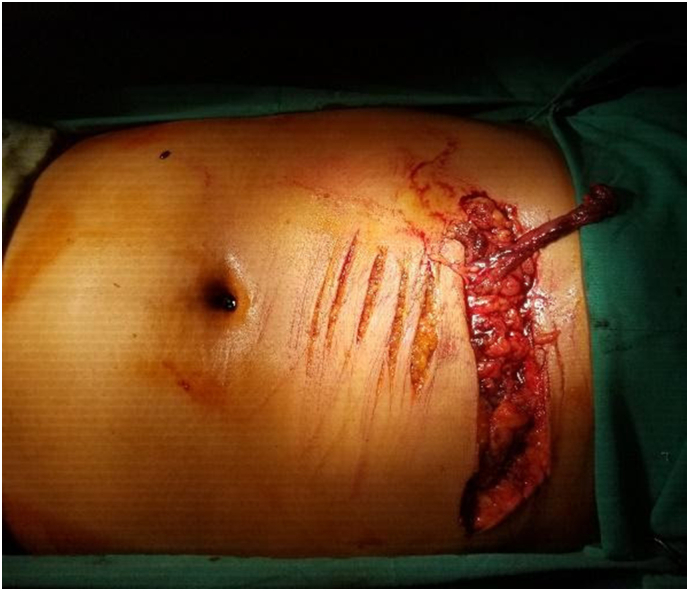
Picture showing multiple transverse hesitation cuts and a single deep penetrating transverse cut with avulsed transverse colon.

**Fig. 2:**
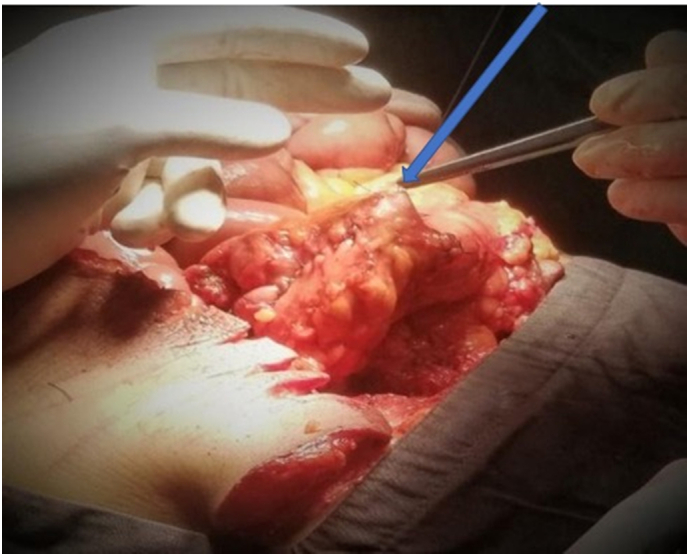
Picture showing colo-colic anastomosis at the time of surgery.

After resuscitation, the patient was transferred to the operating room within fourty-five minutes since injury and abdomen was explored through a midline incision. Intra-operatively, intraperitoneal hematoma with avulsion of a large portion of the greater omentum and missing segment of the mid transverse colon was observed (Fig. 1). However, no solid organ injuries were noted. Thorough peritoneal lavage was done, all omental vessels were suture ligated, either margins of the transverse colon refreshed followed by side-to-side colo-colic anastomosis after mobilizing the left and right colon (Fig. 2). A diversion ileostomy was made and the abdomen was closed with a drain in the pelvis.

Her post-operative period was uneventful and she was discharged on the 9th postoperative day. She was on her regular follow-up with the surgical and psychiatry department and later underwent ileostomy closure after 3 months of the primary surgery.

## 3 Discussion

Despite low mortality rates, self-inflicted injuries can be associated with significant morbidities and they mostly occur in the background of mental illness. A study by Arpi et al. reports the most common method for attempted suicide and self-inflicted injury to be poisoning (68%) and cutting or piercing (20%) [6]. An extensive study by Herpertz et al. describes common methods of self-harm in psychiatric patients to be skin cutting, head and limb banging, skin burning, and severe skin scratching [7]. Careful monitoring of these patients and restricting access to means of self-harm are of paramount importance.

It is important to recognize alarming features of suicidal thoughts and ideas, death wishes and plans, accessibility to means, and methods of committing suicides that apprehend a tendency to attempt suicide. A history of self-infliction or prior suicidal attempts amongst these individuals could be present. This can predict those who are at possible risk of committing suicide or inflicting severe injuries to themselves. In our case, there was no history of previous suicidal attempts or self-harm and our patient had been compliant with her psychiatric treatment. Moreover, the clinicians tried to evaluate the cause of self-infliction and the stressor that led to trauma and our patient admitted to stabbing herself but did not know as to why she did it. They ruled out concurrent hallucinatory experience that could have possibly enacted the act of injury.

A review on self-inflicted injuries by David et al. reported the requirement of surgical management for nearly 75% of self-inflicted wounds [8]. Primary repair can be performed for injuries involving <50% of the colonic wall, however, injuries involving >50% of the colonic wall or extensive mesenteric injuries require resection with anastomosis. Fecal diversion should be considered as a damage control surgery in critically ill patients [9].

Patients sustaining penetrating colon injury without signs of shock, significant hemorrhage, severe contamination, or delay to surgical intervention can be managed with colon repair or resection with anastomosis rather than routine colostomy.

## 4 Conclusions

Safekeeping and supervision of psychotic patients are of utmost importance, and mandates clinicians and caregivers to be more vigilant. However, the question remains unanswered as to what stressors lead to such life-threatening injuries in these patients who are compliant with medications and otherwise stable.

## Declaration of competing interest

Nothing to state.
